# Nuclear Progestin Receptor Phosphorylation by Cdk9 Is Required for the Expression of Mmp15, a Protease Indispensable for Ovulation in Medaka

**DOI:** 10.3390/cells8030215

**Published:** 2019-03-04

**Authors:** Katsueki Ogiwara, Takayuki Takahashi

**Affiliations:** Laboratory of Reproductive and Developmental Biology, Faculty of Science, Hokkaido University, Sapporo 060–0810, Japan; kogi@sci.hokudai.ac.jp

**Keywords:** medaka, ovulation, extracellular matrix degradation, cyclin-dependent kinase 9, Ccni, matrix metalloproteinase 15 expression, nuclear progestin receptor phosphorylation

## Abstract

Ovulation denotes the discharge of fertilizable oocytes from ovarian follicles. Follicle rupture during ovulation requires extracellular matrix (ECM) degradation at the apex of the follicle. In the teleost medaka, an excellent model for vertebrate ovulation studies, LH-inducible matrix metalloproteinase 15 (Mmp15) plays a critical role during rupture. In this study, we found that follicle ovulation was inhibited not only by roscovitine, the cyclin-dependent protein kinase (CDK) inhibitor, but also by CDK9-inhibitor II, a specific CDK9 inhibitor. Inhibition of follicle ovulation by the inhibitors was accompanied by the suppression of Mmp15 expression in the follicle. In follicles treated with the inhibitors, the formation of the phosphorylated nuclear progestin receptor (Pgr) was inhibited. Roscovitine treatment caused a reduction in the binding of Pgr to the promoter region of *mmp15*. The expression of Cdk9 and cyclin I (Ccni), and their association in the follicle was demonstrated, suggesting that Cdk9 and Ccni may be involved in the phosphorylation of Pgr in vivo. LH-induced follicular expression of *ccni*/Ccni was also shown. This study is the first to report the involvement of CDK in ECM degradation during ovulation in a vertebrate species.

## 1. Introduction

Vertebrate ovaries represent a vigorously dynamic structure due to the constant change in follicles over time, and such structural changes in the ovaries are closely associated with remodeling of the extracellular matrix (ECM). In particular, ECM remodeling underlies major changes during ovulation, which denotes the shedding of one or more viable oocytes from fully grown ovarian follicles into the reproductive tract. Ovulation is triggered by a surge in gonadotropin luteinizing hormone (LH) in all vertebrates [1,2,3,4]. It is widely recognized that follicle rupture during vertebrate ovulation involves proteolytic degradation at the apex of ovulating follicles. Using the teleost medaka, a suitable model for ovulation studies, proteolytic processes accompanying follicle rupture during ovulation have been explored [5]. Our previous studies demonstrated that activation of the plasminogen activator/plasmin (Plau/Plasmin) system and matrix metalloproteinase (Mmp) system is required for the hydrolysis of ECM proteins present in the follicle layers of ovulating follicles [6,7,8,9]. Upon activation of the Plau/Plasmin system, active plasmin is generated by proteolytic processing of the liver-derived precursor plasminogen by the follicle-produced active urokinase-type plasminogen activator-1 (Plau1). Plasmin, thus generated, is responsible for hydrolyzing laminin, a major ECM component of the basement membrane [7,8]. After the Plau/Plasmin system is kept active for several hours, another proteolytic system involving three distinct Mmp enzymes, membrane type 1-Mmp (Mt1-mmp/Mmp14), membrane type 2-Mmp (Mt2-mmp/Mmp15), and gelatinase A (Mmp2), becomes active. Mmp2 activated proteolytically by Mmp14 hydrolyzes type IV collagen, another principal component of the basement membrane, while Mmp15 degrades the type I collagen most abundantly present in the theca cell layer [6,9]. During these hydrolytic processes, plasminogen activator inhibitor-1 [8] and the tissue inhibitor of metalloproteinase-2b (Timp2b) [6] are involved in regulating the Plau/Plasmin system and Mmp system, respectively. Among the proteases involved in follicle layer ECM hydrolysis, Mmp15 is the only LH-inducible enzyme. Our recent study demonstrated that LH-induced expression of the *mmp15* gene is accomplished in two steps. In the first step, nuclear progestin receptor Pgr is induced by the LH surge, and the resulting Pgr is then complexed with 17α, 20β-dihydroxy-4-pregnen-3-one (17,20βP)—the physiological progestin ligand for medaka Pgr [10,11,12]—to become an active transcription factor. In the second step, activated Pgr, together with the transcription factor CCAAT/enhancer-binding protein β (Cebpb), contributes to the expression of *mmp15* mRNA [13].

In our attempt to search for genes/proteins involved in the expression of *mmp15* mRNA, we found that the cyclin-dependent protein kinase (CDK) inhibitor roscovitine inhibited not only follicle ovulation, but also the follicular expression of *mmp15* mRNA in the medaka, implicating CDK in the expression of the protease gene in the follicle that is destined to ovulate. In this study, we suggest that after phosphorylation, Pgr becomes a functional transcription factor for *mmp15* gene expression, and that Cdk9 and cyclin I (Ccni) are involved in the process of Pgr phosphorylation.

## 2. Materials and Methods

### 2.1. Animals and Tissues

Adult orange-red variety of medaka, *Oryzias latipes* (himedaka) were purchased from a local dealer and used for the experiments. The fish were maintained in aquariums under an illumination cycle, 14 h of light and 10 h of dark, at 26–27 °C [14], and were fed 3–4 times a day with commercial fish diet (Otohime, Nisshin Co. Tokyo, Japan). Fish under artificial lighting conditions ovulated every day around the transition time from dark to light period. Ovulation hour 0 was set to the start of the light period. Ovaries, ovarian follicles, follicle layers of the follicles, and oocytes were isolated from spawning female fish as previously described [14]. Animal cultures and experimentation were conducted in accordance with the guidelines for animal experiments of Hokkaido University and were approved by the Committee of Experimental Plants and Animals, Hokkaido University (16-0072).

### 2.2. In Vitro Culture of Isolated Follicles

**Figure 1:**
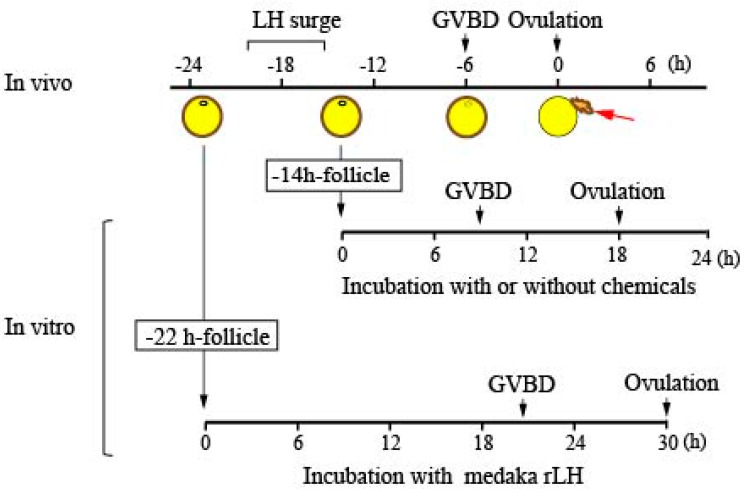
An outline of the in vitro culture experiments using medaka preovulatory follicles. Large follicles destined to ovulate were isolated 22 h before ovulation (−22 h-follicle) or 14 h before ovulation (−14 h-follicle). The follicles were incubated in medium containing medaka recombinant luteinizing hormone (rLH) (for the −22 h-follicle) or without LH (for the −14 h-follicle). Various chemicals were tested in culture using the −14 h-follicles to assess their effects on ovulation, oocyte maturation, as well as gene and protein expression.

In female medaka with a 24-h spawning cycle, postvitellogenic follicles undergo an LH surge approximately 18 h before ovulation and germinal vesicle breakdown (GVBD), an important milestone for oocyte maturation, which occurs 6 h before ovulation in vivo. A procedure for in vitro ovulation using postvitellogenic follicles was established [5]. In the current study, follicle cultures were conducted in 4 mL of 90% M199 medium containing 50 μM gentamycin (pH 7.4), using follicles isolated either 22 h before ovulation (designated as the −22 h-follicle) or 14 h before ovulation (designated as the −14 h-follicle). For the −22 h-follicles, which had not yet been exposed to the in vivo surge of LH, 100 µg/mL medaka recombinant LH (rLH) was included in the culture medium to initiate a series of ovulatory reactions in the follicles. The −14 h-follicles were incubated without medaka rLH, but with various other chemicals, because they had already been exposed to the ovulatory LH surge in vivo. The chemicals used were roscovitine (Merck Millipore, Billerica, MA, USA), MEK inhibitor PD98059 (Merck Millipore), CDK9 inhibitor II (Merck Millipore), and RU486 (also known as mifepristone, Sigma-Aldrich, St. Louis, MO, USA). Compared to the in vivo situation, GVBD and follicle ovulation take more hours under the in vitro culture. Rates of GVBD and ovulation in the incubated follicles were assessed. In addition, expression levels of various genes/proteins in follicles or follicle layers of the follicles were determined. An outline of the in vitro follicle culture used in this study is shown in Figure 1. Follicles were obtained from two to three fish ovaries, pooled, and then divided into control and test groups. The number of follicles per group was approximately 20–25. The duration of incubation and time points at which follicles and/or follicle layers were collected for target gene expression analysis are indicated in the text. Medaka rLH was produced in Chinese hamster ovary k-1 cells as previously described [14].

### 2.3. cDNA Cloning for ccni

cDNA cloning and the sequencing were conducted for medaka *ccni* because the cDNA sequence was not available from public databases. It was determined by PCR using a KOD FX Neo-DNA polymerase (Toyobo, Osaka, Japan) and medaka ovary cDNA. The primers used were Cyclin I 5′-SS and Cyclin I 3′-AS (Table S1). The amplified products were phosphorylated, gel-purified, inserted into the pBluescript II vector (Agilent Technologies, Santa Clara, CA, USA), and sequenced. The sequence determined was deposited into the DDBJ/GenBank/NCBI database (accession number LC435346).

### 2.4. RNA Isolation, Reverse Transcription (RT), and Real-Time Polymerase Chain Reaction (PCR)

Total RNA isolation, RT, and real-time RT-PCR were conducted as previously described [13]. Eukaryotic translation elongation factor 1 alpha 1 (*eef1a1*) was used as a reference gene to normalize the expression of the target genes examined. Relative expression levels were expressed compared to the appropriate controls. The primers used in this study are listed in Table S1.

### 2.5. Preparation of Antigens and Antibodies

RT-PCR was performed to amplify cDNAs encoding medaka Cdk1 (303 residues), Cdk9 (393 residues), Ccni (342 residues), and Ribosomal protein L7 (Rpl7; 245 residues) using KOD Neo DNA polymerase (Toyobo, Tokyo, Japan) with ovary cDNA. The primers used are listed in Supplementary Table S1. The amplified products were phosphorylated and inserted into the prokaryotic expression vector pET30a (Novagen, Madison, WI, USA), which had been previously digested by *Eco*RV. Expression, purification, and dialysis of the protein were performed according to previously published methods [15]. Anti-medaka Cdk1, Cdk9, Ccni, and Rpl7 antibodies were generated using mice according to methods described previously [14]. Mouse anti-medaka Pgr antibody [16], rat anti-medaka Pgr antibody [16], and rabbit anti-medaka Mmp15 antibody [6] were prepared as described previously. Antibodies were purified as previously described [7], and the resulting purified antibodies were used in the experiments.

### 2.6. Immunoprecipitation and Western Blot Analysis

Immunoprecipitation/western blot analysis for Pgr was performed as previously described [12], except that an anti-mouse IgG, HRP-Linked Whole Ab Sheep, was used as a secondary antibody. Immunoprecipitation/western blot analysis for Cdk1, Cdk9, and Ccni was performed in a manner similar to that for Pgr, as described above. Detection of medaka Mmp15 [6], Pgr [16], and Cebpb [13] proteins in follicle layer extracts was performed as previously described. For the detection of Cdk1 and Cdk9 protein, western blot analysis was performed according to the previous method described in Reference [6]. Rpl7 was used as loading control. 

### 2.7. Phosphatase Treatment

The materials immunoprecipitated using rat anti-medaka Pgr antibody were incubated with or without Lambda Protein Phosphatase (New England BioLabs Inc., Ipswich, MA, USA) for 60 min at 30 °C in 1 × NEB Buffer for Protein Metallo Phosphatase supplemented with 1 mM MnCl_2_ and gentle agitation. Incubation was terminated by adding 1 × SDS sample buffer and boiling for 10 min.

### 2.8. Primary Culture of Medaka Granulosa Cells (GC) 

Isolation and primary culture of medaka GCs were performed as previously described [17], except that GCs were isolated from preovulatory follicles, 7 h before ovulation. After culturing for 24 h, the cells were harvested, and the expression of target mRNAs was examined by real-time RT-PCR. 

### 2.9. Digestion of ECM Proteins by Medaka Recombinant Mmp15 

Five micrograms of bovine type I collagen (Sigma-aldrich, St. Loui, MO, USA), bovine type IV collagen, human fibronectin (Sigma), human laminin (Sigma), porcine gelatin (Difco Laboratories, Inc., Detroit, MI, USA), and medaka collagen type I were incubated in 50 mM Tris-HCl buffer (pH 7.5) containing 5 mM CaCl_2_ and 50 μM ZnSO_4_ with medaka recombinant Mmp15 (100 ng) for 16 h at 27 °C. After incubation, reactions were terminated by adding 1 × SDS sample buffer and boiling for 10 min. The resulting samples were subjected to sodium dodecyl sulfate-polyacrylamide gel electrophoresis (SDS-PAGE). After electrophoresis, the gel was visualized by Coomassie brilliant blue R-250 (CBB) staining. Medaka recombinant Mmp15 preparation, active site titration of the enzyme, and purification of medaka collagen type I were performed as previously described [6].

### 2.10. Culturing Cells Stably Expressing Medaka Pgr

Establishment of a cell line stably expressing medaka Pgr [13] using OLHNI-2 cells [16] was performed according to the previous method. The cells were treated for 24 h with or without 17,20βP (100 nM) and/or roscovitine (50 μM). After the treatment, cells were harvested, and the expression of *mmp15* mRNA was examined by real-time RT-PCR. 

### 2.11. Chromatin Immunoprecipitation (ChIP)

ChIP assays were performed according to the methods previously described [13], except that cultured follicles were analyzed in this study. Briefly, the −14 h-follicles were incubated with or without roscovitine and the resulting follicles were used. Preparation of follicle cell layer fractions, sonication, immunoprecipitation with protein G-Sepharose-coupled medaka Pgr antibody, elution, reverse-crosslinking, and real time PCR were carried out as previously described [13]. Eight sets of primers were used, and are listed in Supplementary Table S1. Putative binding sites of Pgr were identified using a free program TFBIND (http://tfbind.hgc.jp/).

### 2.12. Detection of Phosphorylated Pgr

Phos-tag SDS-PAGE using a 7% polyacrylamide gel containing 50 μM Phos-tag^®^ acrylamide (Wako Chemicals, Osaka, Japan) and 50 μM MnCl_2_ was carried out according to the manufacturer’s instructions. Briefly, materials immunoprecipitated with rat anti-medaka Pgr antibody were subjected to Phos-tag SDS-PAGE. After electrophoresis, the gel was incubated twice in a buffer (0.2 M glycine, 20 mM Tris, 0.1% SDS, and 20% methanol) containing 10 mM EDTA, followed by a buffer without EDTA. Transfer to the Immobilon PVDF membranes (Merck Millipore), blocking, and detection of Pgr were performed. 

### 2.13. Immunohistochemistry

Immunohistochemistry was performed according to the method previously described in [8], except that the signal was detected using an ImmPACT AMEC Red Peroxidase (HRP) Substrate (Vector Laboratories, Burlingame, CA, USA), according to the manufacturer’s instructions.

### 2.14. Knockout Experiments for cdk9 

The Cas9 nuclease expression vector was generated as follows. The hygromycin B resistance gene cassette was prepared by PCR amplification using primer pair Hyg SS and Hyg AS (Table S1). pGloSensor™-22F cAMP Plasmid (Promega corporation, Madison, Germany) was used as the template. The PCR product was phosphorylated and inserted into pCS2+hSpCas9 (Addgene Plasmid 51815), which had been digested with Mfe I, filled in by Klenow fragment, and dephosphorylated. The resultant vector, pCS2+hSpCas9/Hyg, was used for knockout experiments. The sgRNA expression vector was generated according to the previous method described in [18]. Briefly, a pair of oligonucleotides was annealed and ligated into pDR274 (Addgene Plasmid 42250), which had been digested with Bsa I. The resultant vector, pDR274-CDK9, was used for KO experiments. A combination of pDR274-CDK9 and pCS2+hSpCas9/Hyg, or pDR274-Mock and pCS2+hSpCas9/Hyg, was co-transfected into OLHNI-2 cells stably expressing medaka Pgr using ScreenFect A (Wako) according to the manufacturer’s instructions. After culture for 48 h, the medium was changed into fresh medium containing 100 μg/mL hygromycin (Wako), and cells were cultured for another 48 h. The cells were harvested and used for the immunoprecipitation/Phos-tag SDS-PAGE analysis. 

### 2.15. Coimmunoprecipitation

Preovulatory follicles isolated from ovaries 7 h before ovulation were sonicated for a few seconds in 50 mM Tris-HCl (pH 8.0) containing a 1 × protease inhibitor cocktail (Wako) and 1× phosphatase inhibitor cocktail (Wako), and then centrifuged at 13,000× *g* for 10 min. The resulting supernatants were used for coimmunoprecipitation analysis. The samples were treated with Protein G-Sepharose (GE Healthcare) that had been previously coupled with anti-medaka Cdk9 antibody. After incubation at 4 °C for 16 h, they were washed four times using 50 mM Tris-HCl (pH 8.0). The precipitant materials were boiled in 1× SDS sample buffer for 10 min and used for western blot analyses.

### 2.16. Statistical Analysis

The experiments performed in this study were repeated independently four to seven times, and all values are presented as the mean ± S.E.M. Statistical significance was verified using Student’s *t*-test or one-way ANOVA followed by Dunnett’s post hoc test, or Kruskal-Wallis test, as appropriate. Equal variation was confirmed by the F-test or Bartlett’s test, as appropriate. The minimum level of statistical significance was set at *p* < 0.05. The experiments for western blot, immunoprecipitation/western blot, and immunohistochemistry analyses were repeated three to five times to confirm their reproducibility, and the results of one experiment are presented.

## 3. Results

### 3.1. Inhibitory Effect of Roscovitine on Medaka Follicle Ovulation

**Figure 2:**
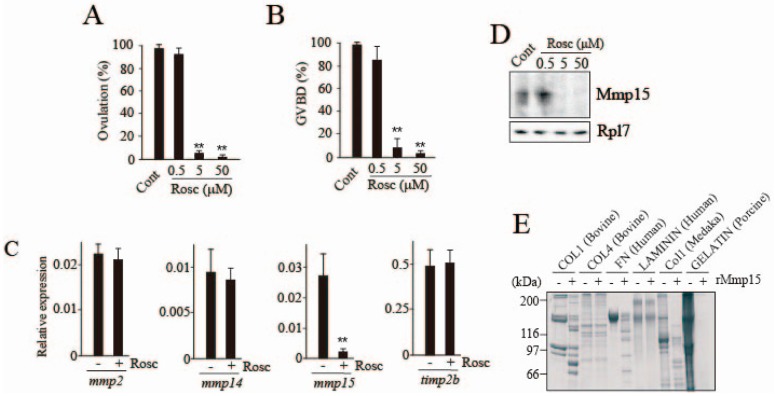
Effects of roscovitine on ovulation, germinal vesicle breakdown (GVBD), and gene expression of ovulation-related Mmps and its intrinsic inhibitor. (**A**) The −14 h-follicles were incubated in vitro with roscovitine (Rosc) at 0.5, 5, and 50 μM for 18 h, and the rate of ovulation was determined. Asterisks indicate significance at *p* < 0.01 (**) compared to follicles incubated without any additives (ANOVA and Dunnett’s post hoc test, N = 5). (**B**) GVBD rate in the follicles was determined. Follicle incubation was conducted as in (**A**), except that the duration of incubation was 14 h. Asterisks indicate a significance at *p* < 0.01 (**) compared to follicles incubated without any additives (ANOVA and Dunnett’s post hoc test, N = 5). (**C**) The −14 h-follicles were incubated with or without Rosc for 18 h, and the expression levels of various Mmps and timp2b were determined by real time RT-PCR. Asterisks indicate significance at *p* < 0.01 (**) (*t*-test, N = 5). (**D**) Follicle layers of follicles that had been incubated with or without Rosc for 18 h were analyzed for Mmp15 expression by western blotting. (**E**) The hydrolyzing activity of medaka recombinant Mmp15 was tested in vitro using various ECM proteins.

The −14 h-follicles spontaneously ovulated in the culture medium in vitro after approximately 20 h, without externally added medaka rLH. Under these conditions, virtually all follicles underwent GVBD within 14 h. However, the follicles failed to ovulate in the medium containing 5 and 50 μM of the CDK inhibitor roscovitine (Figure 2A). Roscovitine also inhibited GVBD (Figure 2B). To determine the viability of the follicles that had been incubated with the inhibitor at 50 μM for 14 h, a trypan blue exclusion test was conducted. The oocyte and follicle cells of the follicles both showed a clear cytoplasm, indicating that the inhibition of ovulation and GVBD in roscovitine-treated follicles was not due to the CDK inhibitor’s toxic effect. Concerning the inhibitory effect of roscovitine on follicle ovulation, we hypothesized that the inhibitor might affect the expression and/or activation of ECM-degrading enzyme(s). Therefore, we examined the effect of roscovitine treatment on the follicular expression of MMPs and their intrinsic inhibitor genes, which are involved in follicle layer ECM degradation at medaka ovulation [6,13]. Transcript levels of *mmp2*, *mmp14*, *mmp15*, and *timp2b* were compared in follicles that had been incubated with or without roscovitine. Among them, *mmp15* expression was drastically and selectively reduced by roscovitine treatment (Figure 2C). Consistent with the result, inhibition of Mmp15 protein expression was observed in follicle layer extracts of roscovitine-treated follicles (Figure 2D). We next examined the in vitro proteolytic activities of medaka recombinant Mmp15 toward several ECM proteins. The enzyme exhibited degrading activities toward bovine and medaka collagen type I, human fibronectin, and porcine gelatin, but not bovine collagen type IV or human laminin (Figure 2E), confirming our previous finding that Mmp15 selectively hydrolyzed collagen type I among the three major ECM proteins (collagen type I, collagen type IV, and laminin) [6,8,19,20]. We further tried to detect the reduction Mmp15 activity using extracts from follicular layers of follicles that had been previously treated with roscovitine. However, this attempt was not successful because the enzyme activity was too low to detect, even with follicle layer extracts of untreated follicles (data not shown).

In summary, the suppression of follicle ovulation by roscovitine treatment appears to be associated with the reduced expression of *mmp15*/Mmp15.

### 3.2. Effects of Roscovitine on Transcription Factors Involved in mmp15 Gene Expression

**Figure 3:**
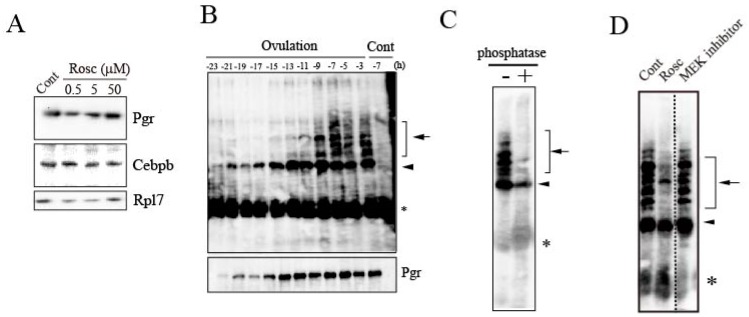
Roscovitine inhibition of Pgr phosphorylation in preovulatory follicles. (**A**) The −14 h-follicles were incubated with roscovitine (Rosc) at 0.5, 5, or 50 μM for 14 h, and the follicle layer extracts were analyzed by western blotting. (**B**) Preovulatory follicles were isolated at various time points, and their extracts were analyzed by immunoprecipitation/Phos-tag SDS-PAGE/western blotting (upper panel) and immunoprecipitation/regular SDS-PAGE/western blotting using an antibody for medaka Pgr (lower panel). Phosphorylated (indicated by arrow) and unphosphorylated forms of Pgr (indicated by arrowhead) are shown in the upper panel. An asterisk indicates the bands corresponding to the antibody used for immunoprecipitation. As controls, the −7 h-follicle extracts were immunoprecipitated using normal IgG. (**C**) The materials immunoprecipitated from follicle extracts obtained from the −7 h-follicles were incubated with or without phosphatase, and the samples were analyzed by Phos-tag SDS-PAGE/western blotting using the antibody for medaka Pgr. Phosphorylated (indicated by arrow) and unphosphorylated forms of Pgr (indicated by arrowhead) are shown. An asterisk indicates the bands corresponding to the antibody used for immunoprecipitation. (**D**) The −14 h-follicles were incubated for 14 h with Rosc (50 μM), with MEK inhibitor (10 μM), or without any additives, and the follicle extracts of the treated follicles were analyzed by immunoprecipitation/Phos-tag SDS-PAGE/western blotting using the antibody for medaka Pgr. Phosphorylated (indicate by arrow) and unphosphorylated forms of Pgr (indicated by arrowhead) are shown. An asterisk indicates the bands corresponding to the antibody used for immunoprecipitation.

We examined the mechanism by which roscovitine inhibited the expression of the *mmp15* gene in preovulatory follicles destined for ovulation. The effects of roscovitine on the follicular expression of two transcription factors, CCAAT/enhancer-binding protein β (Cebpb) and classical nuclear progestin receptor (Pgr), were examined, based on previous knowledge demonstrating that both factors play critical roles in the expression of *mmp15* [13]. Treating the −14 h follicles with roscovitine had no effect on the transcription factors’ expression at the protein level (Figure 3A). Next, we examined the effect of roscovitine treatment on the phosphorylation status of Pgr. In regular SDS-PAGE of untreated follicles, Pgr synthesized in follicles was detected as a single polypeptide throughout the 24-h spawning cycle (Figure 3B, lower panel). This result is consistent with our previous observation on the temporal expression profile of Pgr in follicles destined to ovulate [13]. Pgr was present at low levels in postvitellogenic follicles, but its robust expression occurred several hours after the LH surge. On the other hand, in Phos-tag SDS-PAGE analysis, a single band corresponding to unphosphorylated Pgr was detected in the first half (-23 to -13 h) of the spawning cycle (arrowhead in Figure 3B, upper panel), but multiple slow-migrating bands appeared at later times in the cycle (arrow in Figure 3B, upper panel). Almost all bands with reduced electrophoretic mobility disappeared when the sample was treated with protein phosphatase prior to electrophoresis (Figure 3C), demonstrating that they are phosphorylated forms of Pgr. Roscovitine treatment of the follicle strongly inhibited the formation of phosphorylated Pgr, with the exception of a few bands (Figure 3D). Incubating the follicles with the MEK inhibitor resulted in no significant change compared to the control. In a separate experiment, we confirmed that the phosphorylation of a medaka MAPK protein (Erk), which corresponds to ERK42/44 in mammalian species, was almost completely blocked when the follicle incubated with the MEK inhibitor was analyzed (data not shown), indicating that the MEK inhibitor was effective in inhibiting MEK.

The above results strongly suggest that Pgr is phosphorylated during the last 10 h of the 24-h spawning cycle in follicles destined to ovulate, and that Pgr phosphorylation could be blocked by roscovitine treatment.

### 3.3. Loss of Pgr Binding Ability to the Promoter Region of the mmp15 Gene in Preovulatory Follicles under the Effect of Roscovitine Treatment 

**Figure 4:**
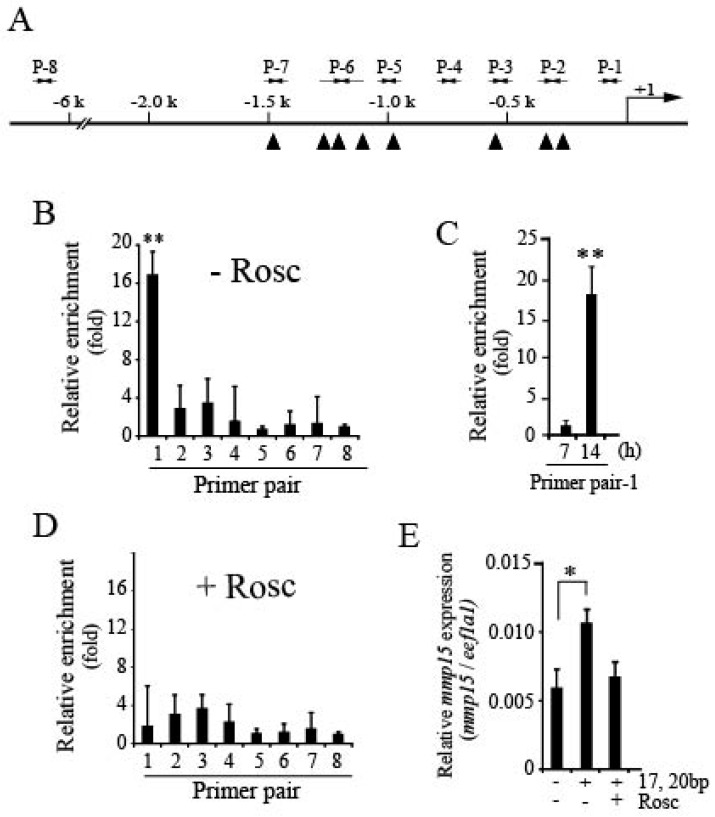
Effect of roscovitine treatment of follicles on Pgr binding to the *mmp15* promoter region. (**A**) For ChIP assays to examine the binding of Pgr to *mmp15* promoter region, seven ChIP primer pairs (P1–P7) in the 1.5 kb upstream region of the transcription start site (indicated as +1) of the *mmp15* gene and another primer pair (P8 as a negative control) were prepared. Putative progestin receptor elements (PREs) in the region are indicated by boxes. (**B**) The −14 h-follicles were incubated in vitro without roscovitine (Rosc) for 14 h, and the resulting follicles were used for ChIP assays and amplified with primer pairs P1 to P8. The sheared DNA immunoprecipitated with anti-medaka Pgr antibody was analyzed by real-time RT-PCR. Asterisks indicate a significant difference at *p* < 0.01 (**) compared to the negative control (ANOVA and Dunnett’s post hoc test, N = 4). (**C**) The −14 h-follicles were incubated in vitro for 7 h or 14 h, and the resulting follicles were used for ChIP assays using primer pair P1. The sheared DNA immunoprecipitated with anti-medaka Pgr antibody was analyzed by real-time RT-PCR. Asterisks indicate a significant difference at *p* < 0.01 (**) compared with the 7 h-incubated follicles (*t*-test, N = 4). (**D**) The −14 h-follicles were incubated in vitro with Rosc (50 μM) for 14 h, and the resulting follicles were used in ChIP assays as in (**B**). (**E**) OLHNI-2 cells stably expressing medaka Pgr were cultured alone, in the presence of 17,20βP (100nM), or in the presence of both 17,20βP and Rosc. After culturing for 24 h, the expression levels of *mmp15* were examined by real-time RT-PCR. Asterisks indicate a significant difference at *p* < 0.05 (*) (ANOVA and Dunnett’s post hoc test, N = 4).

We recently reported that Pgr is an important transcription factor necessary for the expression of *mmp15*, and that it binds to the promoter region of the Mmp gene in granulosa cells of follicles undergoing ovulation [13]. Therefore, the effect of roscovitine treatment on Pgr binding to the *mmp15* promoter in ovulating follicles was examined by performing the ChIP assay (Figure 4A) and using eight primer pairs, as previously reported [12]. First, −14 h-follicles were incubated for 14 h without roscovitine (DMSO only as control) and then analyzed. Among them, primer pair-1, which was designed to generate 101-bp nucleotides corresponding to the sequence between −101 and −1 upstream of the transcription start site of the *mmp15* gene, was only effective for amplifying an expected size of nucleotides (Figure 4B). In this experiment, significant enrichment was not observed with primer pair-2, which—as reported in our previous study [13]—was also effective for the amplification of nucleotides, although the extent of amplification was not as great as that of primer pair-1. The inconsistency in the results of primer pair-2 between our previous and present studies is not clear. In the previous study, we performed ChIP assays using intact follicles isolated from ovaries, whereas in the present study, the assay was conducted using cultured follicles, which might be a reason for the inconsistency. Furthermore, -14 h follicles were incubated with DMSO alone for 7 or 14 h and analyzed using primer pair-1. Pgr recruitment to the promoter was observed for follicles incubated for 14 h (Figure 4C). Next, we examined the effect of roscovitine treatment of follicles on Pgr binding to the promoter region of the *mmp15* gene using eight primer pairs. No significant amplification of nucleotides was found (Figure 4D). Finally, we examined the effect of roscovitine on the 17,20βP-stimulated expression of *mmp15* using OLHNI-2 cells, a cell line established using cells that originated from the medaka fin (Figure 4E). When the cells stably expressing Pgr were treated with 17,20βP for 24 h, *mmp15* expression was significantly increased, confirming that the transcription factor Pgr becomes active by associating with 17,20βP. However, the 17,20βP-induced expression was nullified by the addition of roscovitine.

The results described above indicate that roscovitine treatment of preovulatory follicles causes the inhibition of Pgr binding to the *mmp15* promoter region.

### 3.4. Expression of CDKs in Preovulatory Follicles of the Medaka Ovary

**Figure 5:**
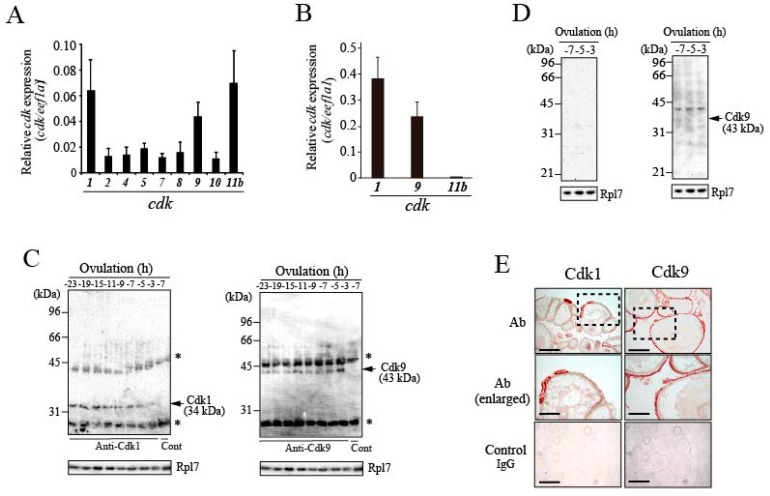
Expression of Cdks in the medaka ovary. (**A**) Relative expression levels of *cdk* transcripts were determined by real-time RT-PCR using total RNAs isolated from follicle layers of preovulatory follicles isolated 6 h before ovulation. (**B**) Relative expression levels of *cdk1, cdk9,* and *cdk11b* transcripts were determined by real-time RT-PCR using total RNAs from granulosa cells isolated from the −6 h-follicles. (**C**) Expression of Cdk1 (left panel) and Cdk9 proteins (right panel) in the preovulatory follicles were analyzed. Extracts from follicles were prepared at various time points in the 24 h spawning cycle and immunoprecipitated with specific Cdk1 and Cdk9 antibodies. The immunoprecipitated materials were then analyzed by western blotting using the same antibodies. Asterisks indicate the bands corresponding to the antibody used for immunoprecipitation. As control, the −7 h-follicle extracts were immunoprecipitated with normal IgG. (**D**) Expression of Cdk1 (left panel) and Cdk9 proteins (right panel) in follicle layer extracts were analyzed. The extracts were prepared using the follicle layers of follicles isolated at 7, 5, or 3 h before ovulation and examined by western blotting using specific antibodies. (**E**) Immunohistochemical analyses were performed in the sections of spawning fish ovaries isolated 15 h before ovulation. Cdk1 (left, upper and middle panels) and Cdk9 (right, upper and middle panels) were stained using the purified antibodies. The area indicated by a box in the upper panels is shown at a higher magnification in the middle panels. Normal IgG was used as a control (lower panels). Bars represent 200 μm in the upper and lower four panels, and 100 μm in the middle two panels.

The results described in the preceding section suggest an involvement of CDK in Pgr phosphorylation in follicles. We therefore examined the expression of CDKs in ovarian follicles of fish ovaries. A computer search for *cdk* genes using the draft medaka genome database (Ensembl genome database: https://asia.ensembl.org/index.html.) revealed that medaka contains a total of 34 *cdk* genes, including *cdk*-like genes. Our recent screening for genes associated with medaka ovulation using next-generation sequencing identified nine *cdk* genes that are presumed to be activated during the ovarian follicle 6 h before ovulation, the time at which Pgr is phosphorylated by Cdk(s). The nine *cdk* genes were indeed expressed in the follicle layer of the -6 h-follicle, as confirmed by real-time RT-PCR; among them, *cdk1*, *cdk9*, and *cdk11b* exhibited relatively high expression (Figure 5A). The levels of the three *cdk* transcripts were not altered in the follicle layers during the 24-h spawning cycle (Figure S1). To examine whether these *cdk* genes are expressed in granulosa cells of preovulatory follicles, real-time RT-PCR analysis was conducted using total RNAs prepared from granulosa cells derived from −6 h-follicles. Amplified products for *cdk1* and *cdk9* were detected (Figure 5B). The expression levels of *cdk11b* mRNA were extremely low, arguing against the idea that Cdk11b might be involved in Pgr phosphorylation in the granulosa cells of follicles.

We next raised specific antibodies for fish Cdk1 and Cdk9 (Figure S2A,B). Single polypeptides of 34 kDa (for Cdk1) and 43 kDa (for Cdk9) were detected by immunoprecipitation/western blot analysis with whole follicle extracts (Figure 5C). The staining intensities of the Cdk1 and Cdk9 bands did not change significantly in the follicles throughout the 24-h spawning cycle. However, follicle layer extracts of follicles predicted to ovulate in 7, 5, and 3 h were analyzed, and Cdk9, but not Cdk1, was detected (Figure 5D). In immunohistochemical analysis, strong signals associated with Cdk1 were observed in the oocyte cytoplasm of small-sized growing follicles (Figure 5E; left panels). Weak signals for Cdk1 were also detected in association with the follicle layers of medium- and large-sized follicles. In addition, a signal was detectable in the oocyte cytoplasm of large-sized follicles. On the other hand, strong signals for Cdk9 were found in the follicle layers of all growing follicles (Figure 5E; right panels).

In vitro treatment of preovulatory follicles with roscovitine did not affect the expression levels of *cdk1*, *cdk9*, and *cdk11b* transcripts in the follicle layer of follicles (Figure S3).

The above results indicate that Cdk9 is the only Cdk species that is constitutively expressed in granulosa cells of ovulating follicles when Pgr undergoes phosphorylation.

### 3.5. Expression of Cyclins in Preovulatory Follicles of the Medaka Ovary

**Figure 6:**
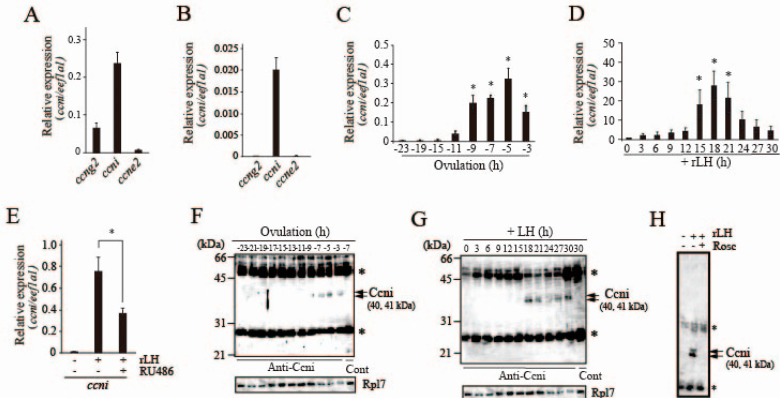
Expression of cyclins in the medaka ovary. (**A**) Relative expression levels of *ccn* transcripts were determined by real-time RT-PCR using total RNAs derived from follicle layers of preovulatory follicles isolated 6 h before ovulation. (**B**) Relative expression levels of *ccng2*, *ccni*, and *ccne2* transcripts were determined by real-time RT-PCR using total RNAs of granulosa cells isolated from the −6 h-follicles. (**C**) Total RNAs were prepared from the follicles at various time points in the 24 h spawning cycle and were used for real-time RT-PCR of *ccni* mRNA. The expression levels were normalized to those of *eef1a1.* Asterisks indicate a significant difference at *p* < 0.05 (*) (Kruskal–Wallis test, N = 5–7). (**D**) The −22 h-follicles were incubated in vitro with or without recombinant medaka LH (100 μg/mL). At various time points of incubation, total RNAs were prepared from follicles and used for real-time RT-PCR of *ccni* mRNA. The expression levels wre normalized to those of *eef1a1* and expressed as the fold change compared to levels of 0 h follicles. Asterisks indicate a significant difference at *p* < 0.05 (*) (Kruskal–Wallis test, N = 4–5). (**E**) The −22 h-follicles were incubated with recombinant medaka LH (100 μg/mL) with or without RU-486 (100 μM). After 18 h of incubation, total RNA was extracted from the follicles and used for real-time RT-PCR analysis of *ccni* mRNA. The expression levels were normalized to those of *eef1a1*. An asterisk indicates a significant difference at *p* < 0.05 (*) (*t*-test, N = 4). (**F**) Expression of Ccni protein in the preovulatory follicles was analyzed. Extracts of the follicles were prepared at various time points in the 24 h spawning cycle and immunoprecipitated using the specific Ccni antibody. The immunoprecipitated materials were then analyzed by western blotting using the same antibody. Asterisks indicate the band corresponding to the antibody used for immunoprecipitation. As a control, the −7 h-follicle extracts were immunoprecipitated with normal IgG. (**G**) The −22 h-follicles were incubated with medaka rLH (100 μg/mL). At various time points of incubation, follicle extracts were prepared and immunoprecipitated with the Ccni antibody. The immunoprecipitated materials were then analyzed by western blotting using the same antibody. Asterisks indicate the band corresponding to the antibody used for immunoprecipitation. As a control, the follicles that had been incubated with medaka rLH for 30 h were immunoprecipitated with normal IgG. (**H**) The −22 h-follicles were incubated with medaka rLH (100 μg/mL) with or without roscovitine (Rosc; 50 μM). After 18 h of incubation, the follicular extracts were immunoprecipitated using the Ccni antibody, and the resultant materials were analyzed by western blotting using the same antibody. An asterisk indicates the bands corresponding to the antibody used for immunoprecipitation.

Cyclins (Ccns) that partner with Cdk for Pgr phosphorylation during medaka ovulation were searched. An Ensembl database search suggested that medaka contains 29 *cyclin* genes, including *cyclin*-like genes. In our attempt to search for ovulation-related genes by next-generation sequencing using preovulatory follicles, three *cyclin* genes, *cyclin G2* (*ccng2*), *cyclin I* (*ccni*), and *cyclin E2* (*ccne2*), were identified as candidates on the basis of FPKM values. Real-time RT-PCR analysis indicated that *ccni* mRNA was most abundantly expressed in −6 h-follicles (Figure 6A). *ccng2* transcripts were also detected in the follicle, but levels were less than one-third those of *ccni*. When granulosa cells were prepared from the −6 h follicle and used for real-time RT-PCR analysis, only *ccni* transcripts were detected (Figure 6B). Therefore, subsequent analysis was conducted with a focus on the *ccni* gene. Time course analysis of *ccni* mRNA levels was investigated using preovulatory follicles isolated from fish ovaries at various time points of the 24-h spawning cycle. The levels of *ccni* mRNA remained low between 23 h and 15 h before ovulation, but *ccni* expression levels increased as ovulation approached (Figure 6C). When the −22 h-preovulatory follicles were cultured in vitro with medaka rLH, the levels of *ccni* mRNA increased (Figure 6D). This rLH-induced *ccni* mRNA expression was significantly reduced by the presence of RU486 (Figure 6E), which was previously shown to act as an antagonist for medaka Pgr [16]. These results suggest that *ccni* mRNA expression in the follicle is regulated by the transcription factor Pgr. To detect Ccni protein in follicles, a specific antibody for medaka Ccni was prepared (Figure S2C). Using the antibody, immunoprecipitation/western blot analysis was performed on whole follicle extracts at various time points. Two bands of approximately 40 and 41 kDa for Ccni were detected between −7 h and −3 h of ovulation (Figure 6F). Similarly, the synthesis of Ccni in the follicle was confirmed between 18 h and 30 h of incubation when the −22 h follicles were cultured with medaka rLH (Figure 6G). These results are consistent with those of the mRNA expression studies described above.

The effect of roscovitine on the follicular expression of Ccni was examined. In vitro roscovitine treatment of −14 h follicles strongly inhibited Ccni expression (Figure 6H), indicating that Cdk may be involved in *ccni*/Ccni expression in the follicle.

The above results showed that follicle cells of follicles destined for ovulation express Ccni, and that its expression is induced by LH. The results also indicate that LH-induced *ccni* expression in the follicle is suppressed by roscovitine treatment.

### 3.6. Possible Involvement of Ccni/Cdk9 in Pgr Phosphorylation in the Preovulatory Follicle

**Figure 7:**
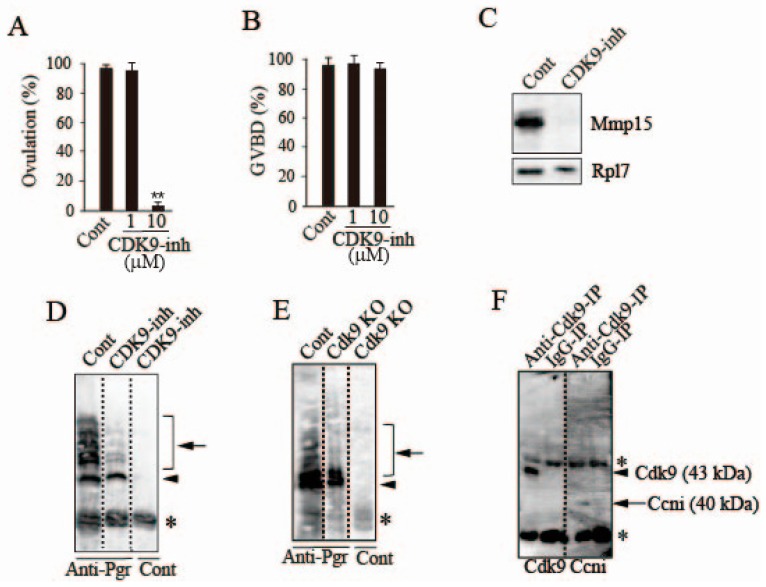
Further evidence for the role of Cdk9/Ccni in the follicles that are destined to ovulate. (**A**) The −14 h-follicles were incubated in vitro with the specific CDK9 inhibitor, CDK9-inhibitor II, at 1 and 10 μM for 18 h, and the rate of ovulation was determined. Asterisks indicate significance at *p* < 0.01 (**) compared to follicles incubated without any additives (ANOVA and Dunnett’s post hoc test, N = 5). (**B**) Incubation of follicles with CDK9-inhibitor II was conducted as in (**A**) except the duration of incubation was 14 h. Note that the inhibitor had no effect on the GVBD of follicles (N = 5). (**C**) The -14 h-follicles were incubated in vitro with or without the specific CDK9 inhibitor CDK9-inhibitor II (10 μM) for 18 h, and the expression of Mmp15 in the follicle layer of the follicles was analyzed by western blotting. (**D**) The −14 h-follicles were incubated in vitro with or without the specific CDK9 inhibitor CDK9-inhibitor II (10 μM) for 14 h, and Pgr phosphorylation in the follicles was examined by immunoprecipitation/Phos-tag SDS-PAGE/western blot analysis. Positions of phosphorylated (indicated by arrow) and unphosphorylated Pgr (indicated by arrowhead) are shown. As control, extracts of the follicles treated with CDK9-inhibitor II were immunoprecipitated with normal IgG. An asterisk indicates the bands corresponding to the antibody used for immunoprecipitation. (**E**) OLHNI-2 cells stably expressing medaka Pgr (Cont) and Cdk9-deficient OLHNI-2 cells (Cdk9 KO), which were generated from the above cells with CRISPR/Cas9 technology, were immunoprecipitated with medaka Pgr antibody, and the resulting precipitated materials were analyzed by Phos-tag SDS-PAGE/western blot analysis. Positions of phosphorylated (indicated by arrow) and unphosphorylated Pgr (indicated by arrowhead) are shown. As a control, extracts of Cdk9-deficient OLHNI-2 cells were immunoprecipitated with normal IgG. An asterisk indicates the bands corresponding to the antibody used for immunoprecipitation. (**F**) Extracts of the −6 h-follicles were immunoprecipitated with medaka Cdk9 antibody or normal IgG, and the resulting precipitated materials were then analyzed by western blotting using the same Cdk9 antibody (left two lanes) or medaka Ccni antibody (right two lanes). Asterisks indicate the bands corresponding to the antibody used for immunoprecipitation.

We examined the effect of CDK9-inhibitor II, a specific inhibitor of Cdk9 [21], on the rate of ovulation using an in vitro follicle culture system. The inhibitor completely suppressed ovulation at 10 μM (Figure 7A). The rate of GVBD was not affected by the inhibitor (Figure 7B), suggesting that Cdk9 is not involved in oocyte maturation in the medaka ovulating follicle. In CDK9 inhibitor II-treated follicles, Mmp15 expression in the follicle layer was strongly suppressed (Figure 7C). To evaluate the possible role of Cdk9 in Pgr phosphorylation, we conducted experiments using follicles cultured with or without CDK9 inhibitor II. In control follicles, phosphorylated Pgr was detected in multiple forms (Figure 7D; Control), but some of the phosphorylated Pgr disappeared or its staining intensity was noticeably reduced after treating the follicles with CDK9-inhibitor II (Figure 7D). A similar result was observed when Cdk9 knockout was generated using the Crispr/Cas9 system in OLHNI-2 cells stably expressing Pgr. In cells transfected with the mock vector, bands for phosphorylated Pgr were detected (Figure 7E; left lane), whereas Pgr phosphorylation was significantly suppressed following Cdk9 knockout (Figure 7E; middle lane). To confirm the reproducibility of the finding, we also conducted experiments using another sgRNA targeting a different Cdk9 sequence, and a similar result was obtained (data not shown), suggesting that the system achieved site-specific DNA recognition and cleavage of the target specifically, without off-target effects. Finally, we examined whether Cdk9 interacts with Ccni in follicles destined to ovulate. Immunoprecipitation was conducted using a Cdk9 antibody with follicle layer extracts of -6 h follicles, and the resulting precipitated materials were further subjected to western blot analysis. The materials contained not only Cdk9 but also Ccni protein (Figure 7F). These results indicate that Ccni and Cdk9 are complexed in follicle cells of follicles undergoing ovulation.

## 4. Discussion

Follicle rupture during ovulation in vertebrates involves well-regulated ECM degradation at the apical region of ovulating follicles. Our previous studies using the teleost medaka showed that two distinct proteolytic enzyme systems, the Plau/plasmin system and the MMP system, contribute to follicle rupture. Among the proteolytic enzymes involved in the process, the role of Mmp15 is of particular interest, in that it serves as a protease that hydrolyzes collagen type I, a major ECM protein in the follicle layer of ovulating follicles, and that its expression is drastically induced in the granulosa cells of follicles at ovulation [6,14]. We recently reported the involvement of at least two transcription factors, Pgr and Cebpb, in the expression of the *mmp15* gene [13]. The current study was initiated following the finding that the CDK inhibitor roscovitine suppressed in vitro follicle ovulation, and the results of this study provide additional information on the regulatory mechanism for *mmp15* expression occurring in the granulosa cells of ovulating follicles. Our new findings are as follows: (i) Pgr is phosphorylated, (ii) the Ccni–Cdk9 complex may be involved in Pgr phosphorylation, and (iii) Ccni is induced by the ovulatory LH surge.

In the present study, we demonstrated that medaka Pgr undergoes phosphorylation prior to its binding to the promoter region of the *mmp15* gene. Electrophoretic analyses of Pgr revealed that the protein is phosphorylated at various sites, as indicated by the appearance of multiple polypeptide bands with a molecular mass greater than that of unphosphorylated Pgr. Our data also suggest that Pgr phosphorylation is largely due to the action of Cdk, because almost all phosphorylated Pgr bands disappeared upon roscovitine treatment of the follicle. Treatment with phosphatase, rescovitine, or CDK9-inhibitor II resulted in no apparent increase in unphosphorylated Pgr. In a separate experiment, we found that the phosphorylated form of Pgr was estimated to be approximately 20% of the total Pgr. Since the proportion of phosphorylated Pgr to total Pgr is low, we presume that changes in the Pgr protein level could not be clearly observed after phosphatase or inhibitor treatment. Previous studies documented that, like medaka Pgr, the human counterpart is also phosphorylated [22,23]. Three PGR isoforms (generally known as PR-A, PR-B, and PR-C) are known in humans, and two major protein kinases involved in human PGR phosphorylation are reported to be MAPK and CDK2. Furthermore, serine residues that are phosphorylated by these protein kinases have been identified. Sequence homologies in fish Pgr and human PGR are very low, hence, a prediction of possible phosphorylation sites in medaka Pgr by searching conserved serine residues between the fish and human proteins was fruitless. Therefore, phosphorylation sites of medaka Pgr were predicted using the free program KinasePhos (http://kinasephos.mbc.nctu.edu.tw/). With this prediction tool, Ser208 and Ser250 were shown to be phosphorylated by Cdk. In addition, Thr14, Thr143, Ser208, and Thr212 could be possible phosphorylation sites for MAPK, although our current data indicated that MAPK has little, if any, effect on Pgr phosphorylation in the fish ovulating follicle. Further studies are needed to establish the validity of such predictions in the future.

We showed that Cdk9 may be responsible for the phosphorylation of Pgr. This idea is supported by the fact that (i) Cdk9 is expressed in the granulosa cells of ovulating follicles, as demonstrated by both immunohistochemical and western blot analyses; (ii) similar to roscovitine, treatment of follicles with the CDK9 inhibitor consistently inhibited follicle ovulation, Pgr phosphorylation, and Mmp15 expression; (iii) Cdk9 knockout experiments using OLHNI-2 cells stably expressing Pgr resulted in a reduction of Pgr phosphorylation; and more importantly, (iv) Cdk9 forms a complex with Ccni in the follicle layers of ovulating follicles. In the context of Pgr phosphorylation, the involvement of Cdk1 is less likely, because the Cdk1 protein is lacking in granulosa cells of ovulating follicles despite the presence of a large abundance of the corresponding mRNA in these cells. Considering that Cdk1 is abundantly detected immunohistochemically in small-growing follicles, Cdk1 may play a role in the early stage of follicle growth. We previously found that, similar to *cdk1*, transcripts of enteropeptidase [15] and melatonin receptor subtype 1c [17] were expressed in the fish ovary, but their translation products were not detectable. Direct evidence for the involvement of CDK9 in Pgr phosphorylation in the granulosa cells of ovulating follicles is needed in future studies.

Interestingly, CDK9-inhibitor II treatment of preovulatory follicles inhibited ovulation, while roscovitine showed inhibitory effects on both ovulation and GVBD of the follicles. This finding indicates that CDKs other than CDK9 may be involved in GVBD, an LH-induced event occurring in the oocyte of medaka.

Ccni was the only cyclin expressed in the granulosa cells of ovulating follicles. Therefore, we tentatively assume that Ccni serves as the regulatory subunit of Cdk9 in the follicle, although Cdk9 activation by Ccni must be experimentally verified. Indeed, we found that Ccni was present in granulosa cells of the follicle in association with Cdk9 protein. Another important finding of the present study is that Ccni is an LH-inducible protein in cells of ovulating follicles. Interestingly, treatment of follicles with RU486, a Pgr antagonist, significantly reduced LH-induced *ccni* expression. This observation strongly suggests that Ccni is expressed in follicles in an LH- and Pgr-dependent manner.

**Figure 8:**
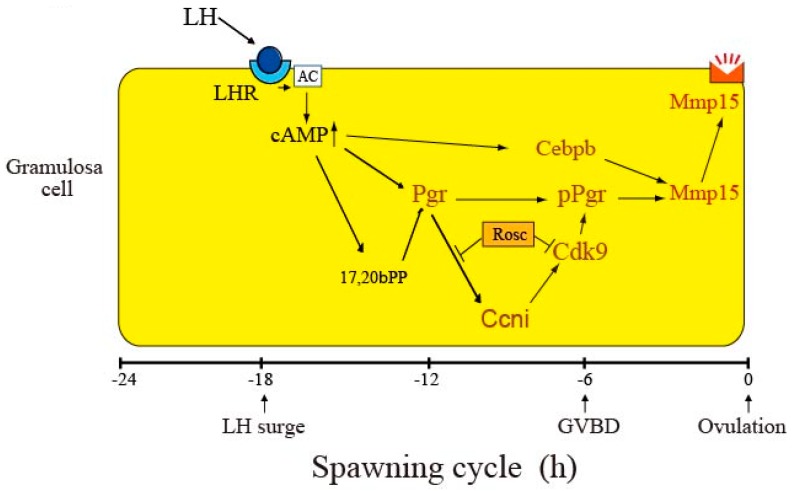
A model for the roles of Ccni and Cdk9 in the process of LH-induced expression of Mmp15 in medaka ovulation. For details, see the text. Note that transcription of *pgr*, *cebpb*, *ccni*, *cdk9*, and *mmp15* gene occurs in the nucleus of the granulosa cell.

Based on the present observations together with our previous findings [6,13], we propose a schematic model for the role of Ccni/Cdk9 in the pathway leading to *mmp15*/Mmp15 expression after the LH surge in follicles (Figure 8). In this model, we focus on the events occurring in granulosa cells, because these cells have been documented to play a major role in fish ovulation [24]. Roscovitine’s effects are also shown in this model. A variety of ovulatory reactions, including the activation of many ovulation-related genes, are evoked in cells expressing the LH receptor in response to the surge of LH, which occurs approximately 18 h before ovulation in the 24-h spawning cycle [14]. Activation of the LH receptor by the LH surge immediately causes an increase in the intracellular cAMP concentration, which leads to the activation of Pgr. Pgr, thus synthesized, would then undergo phosphorylation at multiple sites, mainly via Cdk9. Cdk9, which is constitutively expressed in granulosa cells, should be activated by Ccni. Ccni is also induced in granulosa cells in response to LH stimulation. Our current data suggest that the expression of Ccni is driven by the 17,20βP-activated transcription factor Pgr, and that Pgr-dependent Ccni expression is initiated approximately 9 h before ovulation. Activated Cdk9 complexed with Ccni is capable of phosphorylating Pgr at various sites. The increase in phosphorylated Pgr levels begins at 9 h before ovulation in the granulosa cells of ovulating follicles, and such a situation lasts thereafter until the time of ovulation. Another transcription factor, Cebpb, which is also induced in an LH-dependent manner, is synthesized in granulosa cells of the follicle [13]. At several hours preceding follicle rupture, both phosphorylated Pgr and Cebpb bind to the promoter region of the *mmp15* gene for activation. The resulting translation product, Mmp15, is eventually expressed on the surface of granulosa cells. To substantiate the above hypothesis, further analyses using siRNA and knockout experiments targeting the *ccni* and *cdk9* genes are necessary.

The present study points to the role of Ccni/Cdk9 in the generation of phosphorylated Pgr, which serves as a functional transcription factor for the expression of *mmp15*/Mmp15. However, as documented in studies using mammalian models, alterations in the transcriptional activities of target genes are some of the roles known for phosphorylated Pgr [22]. This posttranslational modification of Pgr may also influence promoter specificity [25], receptor turnover [26], and nuclear association [27]. Further studies are needed to comprehensively clarify the roles of phosphorylated Pgr in the follicle rupture process involving Mmp15 in medaka ovulation.

Another intriguing observation of the present study is that LH-induced Ccni synthesis itself was inhibited by roscovitine. This suggests the involvement of Cdk in the expression of Ccni in granulosa cells of ovulating follicles. Cdk9 is the only Cdk detectable in granulosa cells throughout the 24-h spawning cycle, therefore, we speculate that Cdk9 may contribute to the expression of Ccni. However, there are two important questions relevant to the LH-induced expression of Ccni: i) what molecular species of Ccn could be an activator of Cdk9? and ii) in which process is activated Cdk involved, i.e., transcriptional or translational events of the *ccni* gene? To solve these questions, further studies are required.

In summary, we showed that Pgr, a transcription factor required for the expression of *mmp15*, undergoes considerable phosphorylation in granulosa cells of ovulating follicles, and that only the phosphorylated form of Pgr likely functions as a potent transcription factor for *mmp15* expression. Our data indicated that Ccni/Cdk9 may be responsible for Pgr phosphorylation. To our knowledge, this is the first study to demonstrate the involvement of Cdk in the process of follicle rupture during ovulation in vertebrates. The findings provided in this study help elucidate the whole process of ECM degradation that occurs in follicles during ovulation after the ovulatory surge of LH.

## Supplementary Materials

The following are available online at https://www.mdpi.com/2073-4409/8/3/215/s1. Figure S1: Expression of *cdk1*, *cdk9*, and *cdk11b* mRNA in medaka preovulatory follicles during 24 h-spawning cycle, Figure S2: Characterization of antibodies prepared in this study, Figure S3: Expression of cdk1, cdk9, and cdk11b mRNA in preovulatory follicles cultured with or without roscovitine, Table S1: PCR primers used in this study.
