# Supplementary material for: Nuclear Progestin Receptor Phosphorylation by Cdk9 Is Required for the Expression of Mmp15, a Protease Indispensable for Ovulation in Medaka
Source: Cells. 2019 Mar 4;8(3):215. doi: 10.3390/cells8030215 (PMC6468418; doi:10.3390/cells8030215)
Supplement: Supplementary file 1 [file cells-08-00215-s001.pdf]

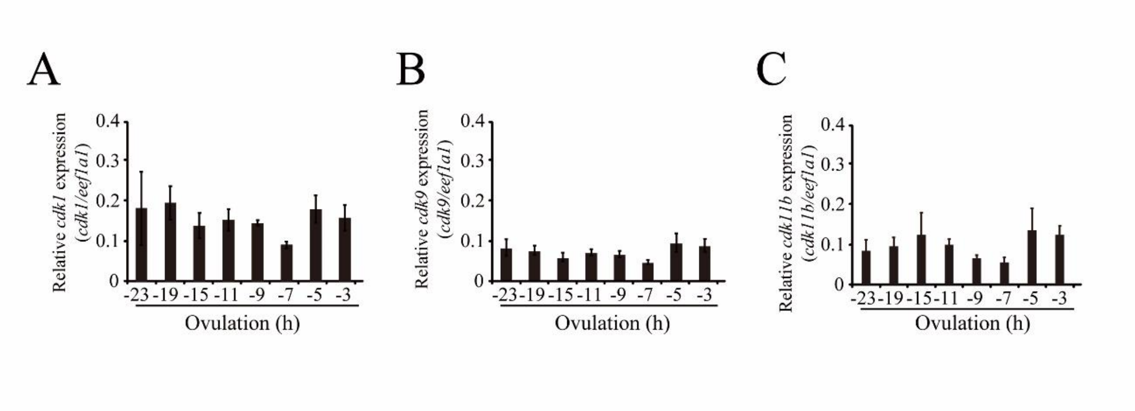

Figure S1. Expression of *cdk1*, *cdk9*, and *cdk11b* mRNA in medaka preovulatory follicles during 24-h spawning cycle.

Total RNAs were prepared from the follicles at various time points in the 24-h spawning cycle and were used for real-time RT-PCR of *cdk1, cdk9,* and *cdk11b* mRNA. Relative follicular expression is shown (N=5-7).

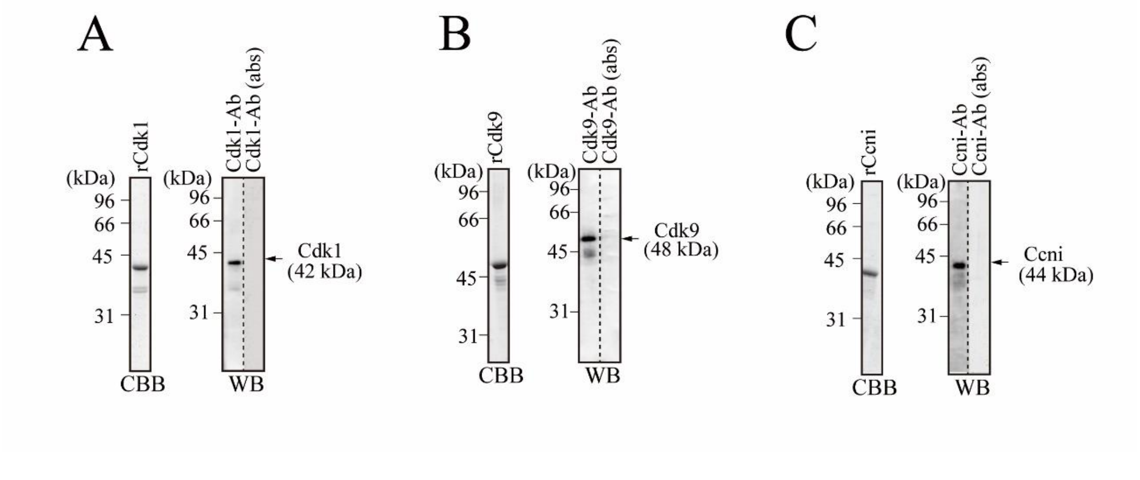

Figure S2. Characterization of antibodies prepared in this study.

Antibodies raised in this study were characterized. Recombinant Cdk1 (A), Cdk9 (B), and cyclin I (C), which were separately used as antigens for immunizing mice, were analyzed for purity by SDS-PAGE/CBB staining (CBB). SDS-PAGE/Western blot analysis was conducted for Cdk antibodies using extracts of ovaries isolated from spawning female fish at 5 h before ovulation (left lane in each panel of WB). As negative control, the antibodies were preincubated with the respective antigens, and the absorbed antibodies (abs) were used (right lane in each panel of WB). Positions of various molecular masses are indicated.

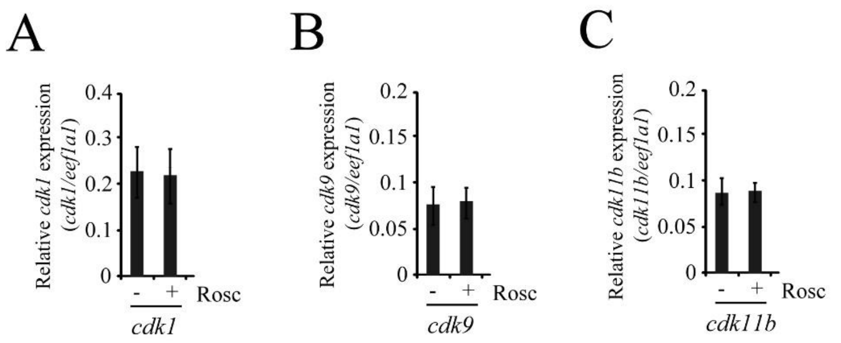

Figure S3. Expression of *cdk1*, *cdk9*, and *cdk11b* mRNA in preovulatory follicles cultured with or without roscovitine.

The -14 h-follicles were incubated with or without Rosc (50 µM) for 18 h, and the expression levels of *cdk1*, *cdk9*, and *cdk11b* were determined by real time RT-PCR (N=5).

Supplemental Table S1. Primers used in this study.

Primer name	gene	Sequence	Accession No.
Real-time PCR			
mmp2 ss	*mmp2*	5′- GCGACGACGGCTTTTTGTGG-3′	AB033754
mmp2 as	*mmp2*	5′- CATCTCCATTCCCTCCCAG-3′	
mmp14 ss	*mmp14*	5′-CGGAGGGTTTCCACGGCGAC-3′	AB185847
mmp14 as	*mmp14*	5′-CCAATCGTCCACGGCTCAGC-3′	
mmp15 ss	*mmp15*	5′-ACGGCTCCCTCCAGTTTTA-3′	AB072928
mmp15 as	*mmp15*	5′-GGTGTTGTTCCTGCGTCTTC-3′	
timp2b ss	*timp2b*	5′-TGGAGACTGATGGGACGATG-3′	AB193468
timp2b as	*timp2b*	5′-GCACGCAGGGAATGGAAGTG-3′	
cdk1 ss	*cdk1*	5′-ATGGAGGACTACGTGAAAATAG-3′	AB040436
cdk1 as	*cdk1*	5′-GGAACTCCCTCCTCCTCACTCT-3′	
cdk2 ss	*cdk2*	5′-ATGGATACGTTTCAGAAAGTGG-3′	XM_020703479
cdk2 as	*cdk2*	5′-CGAATCGCTGTGCTTGGTACGC-3′	
cdk4 ss	*cdk4*	5′-ATGGCGCAGTGCACCGGTCTGC-3′	XM_011475449
cdk4 as	*cdk4*	5′-CGCGCACGTTCTTCAGGGCGAC-3′	
cdk5 ss	*cdk5*	5′-ATGCAGAAATATGAAAAGCTTG-3′	XM_004079396
cdk5 as	*cdk5*	5′-TCGTCATCGTCTAGTCTGACTC-3′	

cdk7 ss	*cdk7*	5′-ATGTCTGTGGATGTGAAAACAA-3′	XM_004072423
cdk7 as	*cdk7*	5′-AATGGCAACTATTGTGTCAGTC-3′	
cdk8 ss	*cdk8*	5′-ATGGACTATGACTTTAAACTGA-3′	XM_004081058
cdk8 as	*cdk8*	5′-GTACACATGACCGTAGGTGCCT-3′	
cdk9 ss	*cdk9*	5′-ATGCAACGAGACAAAACAAGCA-3′	XM_004086584
cdk9 as	*cdk9*	5′-TCGTACTTGGAGAACTCGTCGC-3′	
cdk10 ss	*cdk10*	5′-ATGGACGCCACTGGAGAGGAAG-3′	XM_011476444
cdk10 as	*cdk10*	5′-ACCTATCCGATTGAGTTTCTCA-3′	
cdk11b ss	*cdk11b*	5′-ATGGAAATAACAATTCGTAATT-3′	XM_023956292
cdk11b as	*cdk11b*	5′-GAGACTTTTCCTTTCTTGCCAC-3′	
cyclin I ss	*ccni*	5′-ATGAAGAGCCCAGGAGCCGCAG-3′	LC435346
cyclin I as	*ccni*	5′-GATGTTGGGAAAAGGAGATGTC-3′	
cyclin G2 ss	*ccng2*	5′-ATGGACGCCGTCAAGCTGATGA-3′	XM_004075148
cyclin G2 as	*ccng2*	5′-CACTTGGCGGAGATCCTGCTGT-3′	
cyclin E2 ss	*ccne2*	5′-ATGTCAAGACGCAGTGGTCGCA-3′	XM_023964550
cyclin E2 as	*ccne2*	5′-CTTGGAGGCAGGCTGGAGCTTC-3′	
ef1a ss	*eef1a1*	5’-CACCGGTCACCTGATCTACA-3’	AB013606
ef1a as	*eef1a1*	5’-GCTCAGCCTTGAGTTTGTCC-3’	
ChIP			
Primer pair-1 SS for mmp15	*mmp15*	5′-GAAAGTCATGACGTCACTGG-3′	
Primer pair-1 AS for mmp15	*mmp15*	5′-GGCTCCGCTCCTCCAGAGCC-3′	
Primer pair-2 SS for mmp15	*mmp15*	5′-GTTAGGTTAGGCACACTTAA-3′	
Primer pair-2 AS for mmp15	*mmp15*	5′-TGGAATGTAAACCATTACAA-3′	
Primer pair-3 SS for mmp15	*mmp15*	5′-CATGTTATAACACATGGATG-3′	
Primer pair-3 AS for mmp15	*mmp15*	5′-TTAAGTGTGCCTAACCTAAC-3′	
Primer pair-4 SS for mmp15	*mmp15*	5′-CCAATGCTAATGGATACGCT-3′	
Primer pair-4 AS for mmp15	*mmp15*	5′-ATGTGAACCACTTTGATGAT-3′	
Primer pair-5 SS for mmp15	*mmp15*	5′-CTAAAGTTTCCCATTAACCT-3′	
Primer pair-5 AS for mmp15	*mmp15*	5′-GTCATATTTCACTCCCACAA-3′	
Primer pair-6 AS for mmp15	*mmp15*	5′-CTTTTGTCACCGTCTCCAT-3′	
Primer pair-6 AS for mmp15	*mmp15*	5′-GACAAGATGGCCACCAGCTT-3′	
Primer pair-7 AS for mmp15	*mmp15*	5′-TTTCGTGCGGATGATATATC-3′	
Primer pair-7 AS for mmp15	*mmp15*	5′-TTGCAGCACATTAAGATTGG-3′	
Primer pair-8 SS for mmp15	*mmp15*	5′- AGATATTGCAGTTGCTATACGG-3′	
Primer pair-8 AS for mmp15	*mmp15*	5′- TCATCTGTGTTCCTCTTTCAC-3′	

**Recombinant proteins**			
Cdk1 pET SS	*cdk1*	5′-ATGGAGGACTACGTGAAAATAG-3′	AB040436
Cdk1 pET AS	*cdk1*	5′-TCAGACGCCGTTGATGCAGGCG-3′	
Cdk9 pET SS	*cdk9*	5′-ATGCAACGAGACAAAACAAGCA-3′	XM_004086584
Cdk9 pET AS	*cdk9*	5′-CTAAAAGACTCGGTCGAACTCC-3′	
Cyclin I pET SS	*ccni*	5′-ATGAAGAGCCCAGGAGCCGCAG-3′	LC435346
Cyclin I pET AS	*ccni*	5′-CTAGTTGACAGCAGGGTGCAAT-3′	
Rpl7 pET SS	*rpl7*	5′-ATGGCGGACGCAGAAAAAAAAG-3′	DQ118296
Rpl7 pET AS	*rpl7*	5′-TTAGTTCATCCTTCGGATCATT-3′	
**Cloning**			
cyclin I 5’-SS	*ccni*	5′-ATCATCTCAATGCTCCGTCCAT-3′	ENSORLT00000001568
cyclin I 3’-AS	*ccni*	5′-TCATTTGACCCTGGAGAGCTTG-3′	
**Crispr/Cas9**			
cdk9-SS for Crispr	*cdk9*	5′-TAGGCTCTGAAGAAAGTGCTGA-3′	XM_004086584
cdk9-AS for Crispr	*cdk9*	5′-AAACTCAGCACTTTCTTCAGAG-3′	
Hyg SS		5′-GCGCAGCACCATGGCCTGAA-3′	
Hyg AS		5′-ACACAAAAAACCAACACACA-3′	
